# Transcriptional profiling and genes involved in acquired thermotolerance in Banana: a non-model crop

**DOI:** 10.1038/s41598-018-27820-4

**Published:** 2018-07-16

**Authors:** S. M. Vidya, H. S. Vijay Kumar, R. M. Bhatt, R. H. Laxman, K. V. Ravishankar

**Affiliations:** 1 Division of Biotechnology, ICAR- Indian Institute of Horticultural Research, Hessaraghatta Lake Post, Bengaluru, 560 089 India; 2 Department of Biotechnology, Centre for Post Graduate Studies, Jain University, Jayanagar, Bengaluru, 560 011 India; 3 Division of Plant Physiology and Biochemistry, ICAR-Indian Institute of Horticultural Research, Hessaraghatta Lake Post, Bengaluru, 560 089 India

## Abstract

Banana is a non- model crop plant, and one of the most important crops in the tropics and sub tropics. Heat stress is the major abiotic stress affecting banana crop production because of its long growth period and is likely to become a threat due to global warming. To understand an acquired thermotolerance phenomenon at the molecular level, the RNA-seq approach was employed by adapting TIR method. A total of 136.38 million high quality reads were assembled. Differentially expressed genes under induction (I) was 3936, I + L was 2268 and lethal stress was 907 compared to control. Gene ontology and DGE analysis showed that genes related to heat shock factors, heat shock proteins, stress associated proteins, ROS scavenging, fatty acid metabolism, protein modification were significantly up regulated during induction, thus preparing the organism or tissue at molecular and cellular level for acquired thermotolerance. KEGG pathway analysis revealed the significant enrichment of pathways involved in protein processing, MAPK signaling and HSPs which indicates that these processes are conserved and involved in thermo tolerance. Thus, this study provides insights into the acquired thermotolerance phenomena in plants especially banana.

## Introduction

Global warming predicts an increase of 2–6 °C in ambient temperature by the end of the 21^st^ century^1^. This increased temperature could be a rate limiting factor affecting crop productivity. Like many organisms, plants have both the inherent ability to survive at high temperatures (basal thermotolerance), and the ability to acquire thermotolerance^2^. Acquired thermotolerance may be induced by either pre-exposure to short, but sub-lethal stress or continuous gradual increase in temperature over a long period. It has been shown in many crop species that a great variability can be observed for any given abiotic stress trait under acquired thermotolerance^3^.

*Musa* species are herbaceous monocots and bear popular fruit, banana. Banana, being the staple food crop in many countries, because of its long crop growth period it experiences a variety of abiotic stresses. However, there are no studies pertaining to heat stress tolerance in banana, and especially for acquired thermotolerance. In order to assess the variability and degree of thermotolerance in crop species, various methods have been employed. One of the widely employed method is the temperature induction response (TIR), where plants are exposed to mild temperatures before being exposed to higher (lethal) temperatures. It has been shown that TIR technique brings about potential thermotolerance of individual genotypes. Thus, it is widely used to study genetic variability for thermotolerance in plants^4^. The TIR method allows us to examine the degree of acquired thermotolerance and the variability in germplasm. This concept of induction is based on slow and continuous increase in the temperature conditions i.e acclimating the plants to a certain temperature above the normal growth conditions. One of the examples widely studied on *A. thaliana* to a pernicious heat stress treatment at 45 °C where the accumulation of some transcripts were correlated with that of the induced seedlings that were found to have massive increase in numbers when compared to the non induced ones. Thus, it is important to study the effect of gradual increase or induction stress on early signaling process and activation of genes leading to the accumulation of heat shock proteins (HSPs) and other metabolites involved in thermotolerance.

The whole genome sequencing^5^ and transcriptome analysis^6^ in Banana have advanced our knowledge on *Musa* genome. RNA-seq and DGE are efficient approaches which help in understanding signaling mechanism, pathways, metabolomics, and novel gene discovery. Further, it enhances our knowledge on thermotolerance at the molecular level. Hence, in this study, we have employed RNA-seq, in a non-model crop, to understand molecular changes during induction of heat stress and its role in imparting acquired thermotolerance. The genes and pathways identified would broaden our understanding and provide a new insight, into acquired thermotolerance phenomena in plants.

## Results and Discussion

### Transcriptome analysis under heat stress

**Figure 1:**
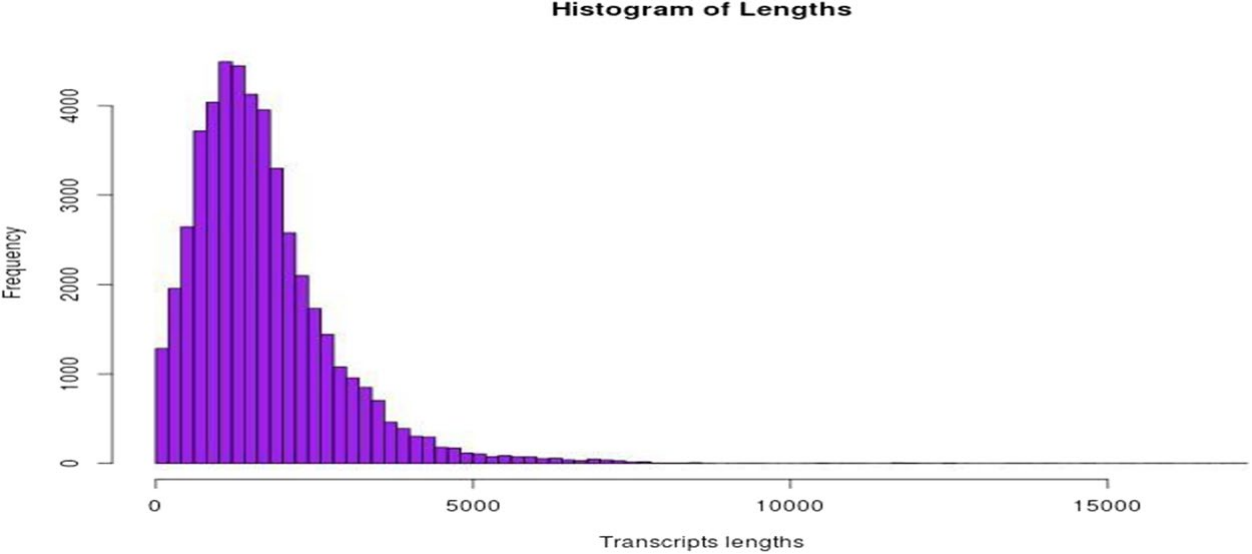
Histogram of distribution of transcript length and number of sequences.

Heat stress is sensed by plants, initially through the leaf at whole plant level^7^, which later brings about physiological and biochemical changes at whole plant level. Leaf samples were harvested from seedlings of cv. Grand Naine at different treatments and total RNA was isolated. Total RNA isolated from three different replicates were pooled for each treatment separately. Untreated or Control, Induction stress (I) where plants had a chance to acclimatize to the heat stress (30 °C to 42 °C for 2 hr 30 min) followed by lethal stress where plants are exposed to high temperature stress conditions (I + L: 55 °C for 2 hr). A set of plants were directly exposed to severe high temperature referred as lethal stress (L: 55 °C for 2 hr). In our previous study, we have observed induced seedlings had lower reduction in growth and better recovery after lethal stress where percent survival was 50% in case of induction and 20% under lethal stress^8^. The RNA from the leaf samples were used to generate four independent libraries and sequenced using the Illumina Next seq. 500 system. Illumina raw reads were processed by an in-house script for removal of adapters and low quality reads. Finally, a total of 136.38 million reads were obtained from four treatments of Control, Induction (I), Induction followed by lethal (I + L), and Lethal stress (L). High quality reads were aligned to the banana reference genome database (DH-Pahang, *Musa acuminata* (AA) genotype^5^: Table 1). The frequency of transcript length vs. the number of sequences is shown in Fig. 1, where higher number of transcripts were observed in the class 200–1000 bp. This is the first transcriptome library to be reported for heat stress in banana, a triploid non-model crop and also for induction stress in plants. Other transcriptome studies so far reported on abiotic stress are on banana root^9^ (for osmotic stress), switchgrass (for heat stress)^10^, grape (for heat stress)^11^ however, none of the studies examined changes during induction stress and its role in acquired thermotolerance. In wheat, microarray analysis of heat stress samples, showed 1525 differentially expressed transcripts^12^. SSH based studies on tall fescue reported 1090 transcripts for high temperature stress^13^. In Barley, short term exposure to heat stress, and later using Gene Chip analysis, identified 10520 probe sets expressed at 12 days post anthesis^14^. A high throughput de novo transcriptome assembly sequencing was carried out for Spinach under heat stress, a total of 33573 unigenes were identified that matched with the public databases^1^. From the present study, transcriptome data has been deposited at NCBI, under the accession SRA (SRP074337:PRJNA320030). We have observed higher number of gene expression in induction followed by I + L. Thus the heat stress considerably alters the gene expressed in banana.

**Table 1:** Total number of raw reads and clean reads that were mapped from four libraries.

Sample	Raw reads	Filtered reads		Total	Mapped	Percentage (%)
		PE	SE			
Control	2*11796089	2*4238581	1578967	10056129	7635750	76
Induction	2*19958655	2*5803050	1105436	12711536	10777643	84.7
I → L (I + L)	2*20473094	2*4702934	7413241	16819109	13243939	78.7
Lethal (only)	2*27014963	2*17281524	1271114	35834162	32220070	89.9

PE: Paired end, SE: Single end.

### Functional annotation and gene ontology analysis of banana heat responsive transcripts

For annotation, RNA-seq data was mined using different databases: http://www.ncbi.nlm.nih.gov/bioproject/320030 NCBI, UniProt: Swiss-Prot and Pfam with an E-value <= 1e^−5^. Out of 69972 transcripts identified, 51556 were annotated to Swiss-Prot database, 66884 annotated to UniRef database, and 52279 annotated to Pfam database.

**Figure 2:**
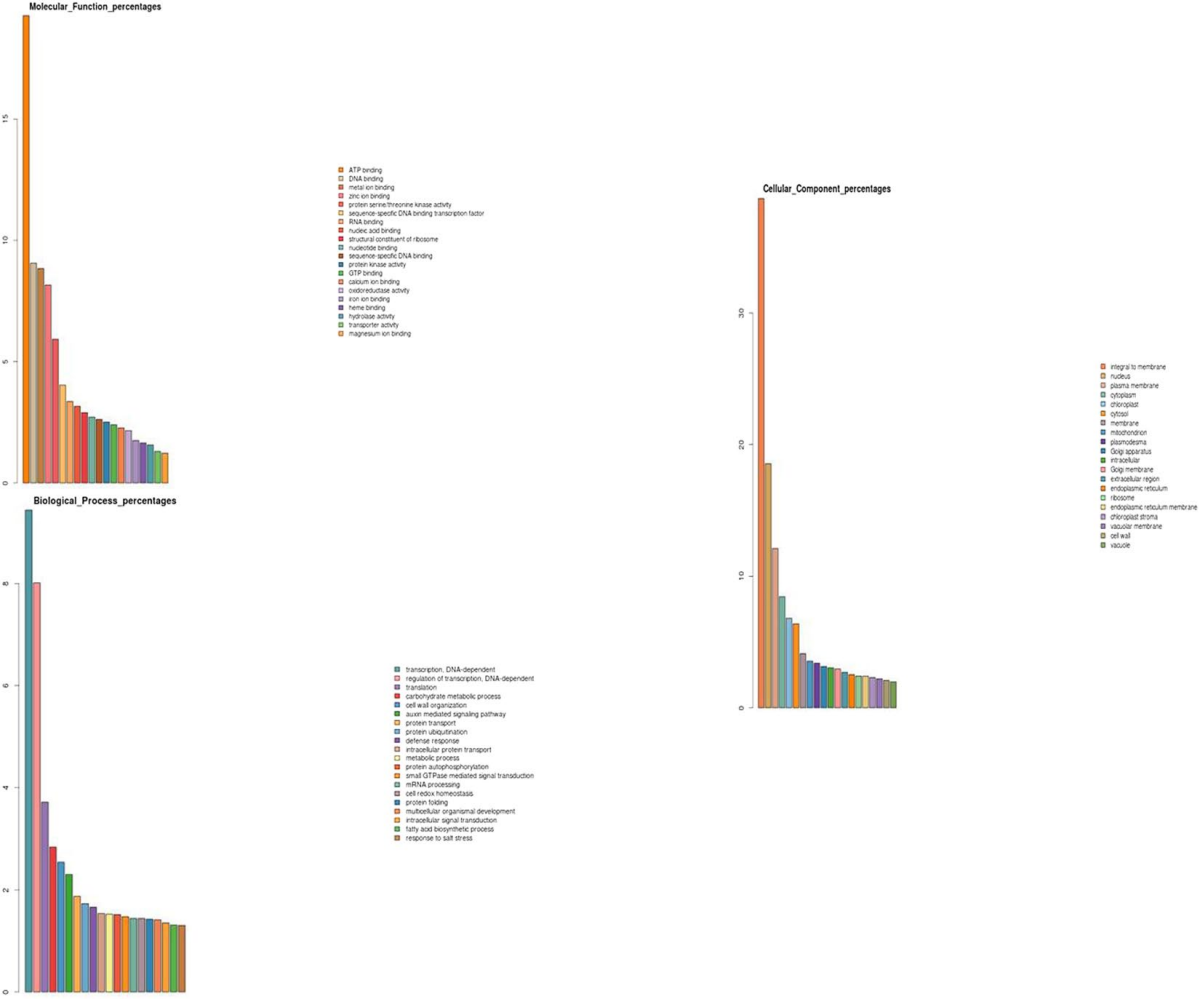
Gene ontology classification of the heat stress transcriptome. (**A**) Molecular function (**B**) Cellular components (**C**) Biological process.

In order to investigate, the genes altered during heat stress, the gene ontology (GO) descriptions were studied. The distribution of the transcripts showed that they belong to three categories namely: molecular functions, biological process, cellular components. Among the heat stressed samples, higher number of genes were found in molecular functions followed by a biological process or biochemical processes, and fewer were involved in regulation of cellular components. These genes were classified based on their roles and functional categories. Under the molecular function category, genes were mostly associated with ATP and DNA binding (Fig. 2A). In biological functions, where most of the transcripts were associated with transcription, translation, and carbohydrate metabolism (Fig. 2B). In cellular components, the majority of the genes were associated with integral membrane, nucleus, other cell components (Fig. 2C). The results of analysis indicated that genes involved in membrane integrity, transcription and translation are highly altered during heat stress. Therefore, apart from membrane integrity, the gene expression and protein synthesis are affected during high temperature stress.

Since, abiotic stress tolerance is polygenic in nature and also governed by many transcription factors^15^, we have employed Annocript software to identify conserved domains using Pfam database (http://pfam.xfam.org/). The following domains were identified, Protein kinase (pfam00069), MYB like DNA binding (pfam13921), HLH domain (pfam00010), WRKY (pfam03106), PPR (pfam13041). In case of lethal stress, HSF DNA-binding protein (pfam00447), HSP20 (pfam00011) and p450 cytochrome (pfam00067) in induction and in case of I + L (induction followed by lethal) stress. All these domains are known to play important roles in signaling, and activation of transcription factors and several heat stress associated proteins during induction, which later impart tolerance during lethal or higher degree of stress^16^.

Further, in the pathway analysis, top hits were transcripts involved in protein modification, lipid metabolism, secondary metabolite biosynthesis, and carbohydrate metabolism. The above pathways, stabilizes the membrane, where many stress associated proteins and various HSPs get activated. HSP70, HSP90, HSP100 family of heat shock proteins are known to act as molecular chaperone to prevent aggregation and denaturation. Thus contributing to protein refolding and maintaining protein structure. In earlier studies, the overexpression of HSP70 has shown its involvement in translation and proper folding of denatured proteins^17,18^. Heat stress induces membrane fluidity which activates the lipid signaling, further affecting the Ca^2+^ channels and antioxidants. The lipid saturation is therefore an important aspect, where increase in saturated fatty acids helps in maintaining the membrane fluidity. These cascades of reactions act as a primary signal for activation of heat stress tolerance^9^.

In a heat stress study conducted on Spinach, the results of KEGG pathways analysis showed 975 unigenes were up-regulated, which were involved in plant abiotic and biotic stress conditions^1^. Several studies have showed that plants activate antioxidant enzymatic systems to avoid the oxidative burst due to abiotic stresses. In the present study, we observed that many antioxidant genes were activated, most common being superoxide dismutase and peroxidase during induction stress (Supplementary File S1). The results of analysis show that the genes involved in signaling, HSP synthesis, antioxidant and lipid biosynthesis are activated during induction and involved in imparting acquired thermotolerance.

### Differential gene expression profiling under heat stress

The differential gene expression at various stages of heat stress treatment were examined, and a comparative analysis was done using the aligned reads. All the unigenes from three heat stressed samples were clustered and compared in the following combinations.

1. Control (untreated) vs Induction stress (I)
2. Control (untreated) vs Induction stress followed by Lethal stress(I + L)
3. Control (untreated) vs Lethal stress (L) (directly exposed to lethal stress)

**Figure 3:**
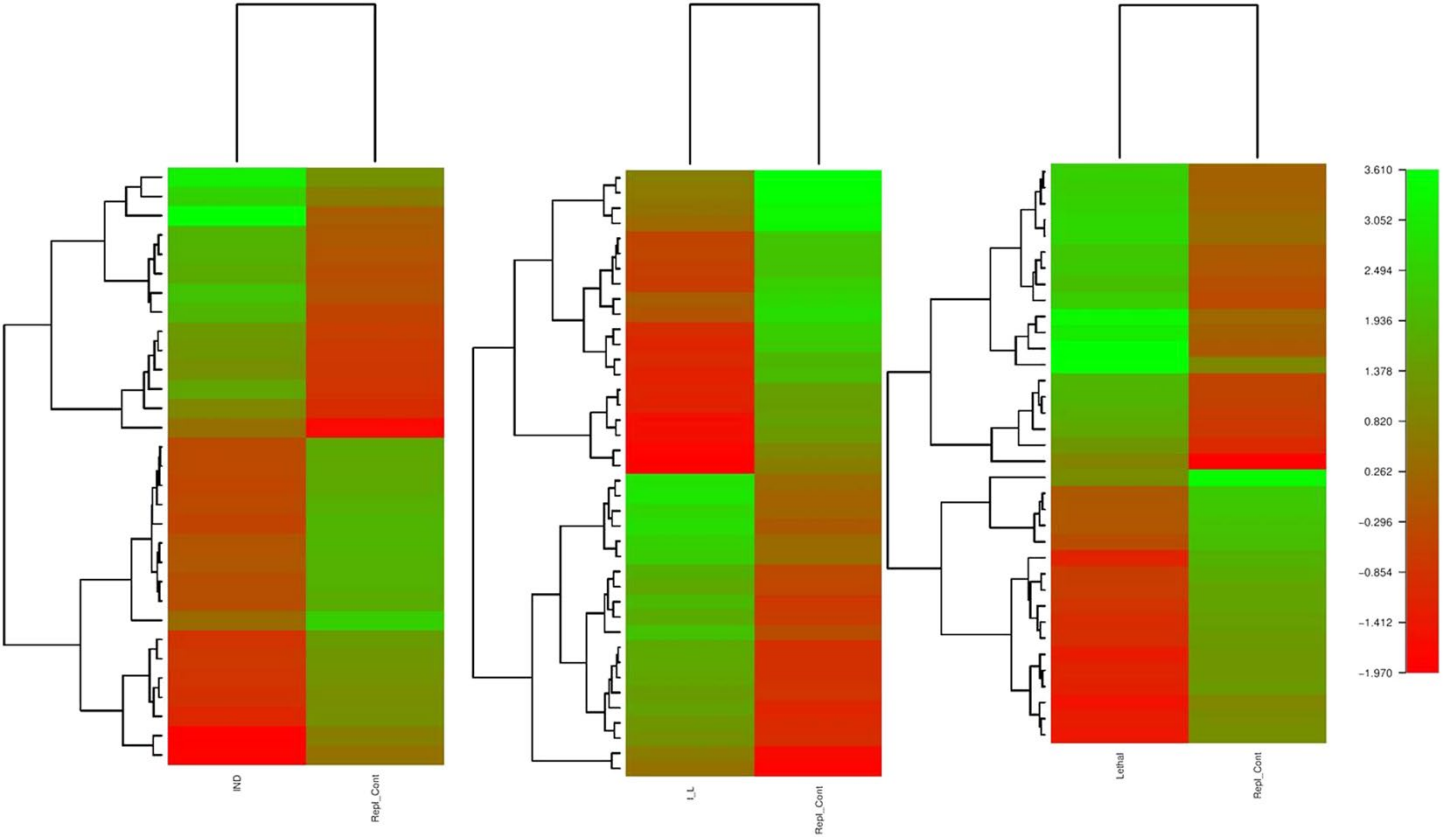
Heat stress profiling of banana under different stress conditions *viz* Induction, Induction followed by lethal, Lethal stress: The heat map was constructed using R package.

**Figure 4:**
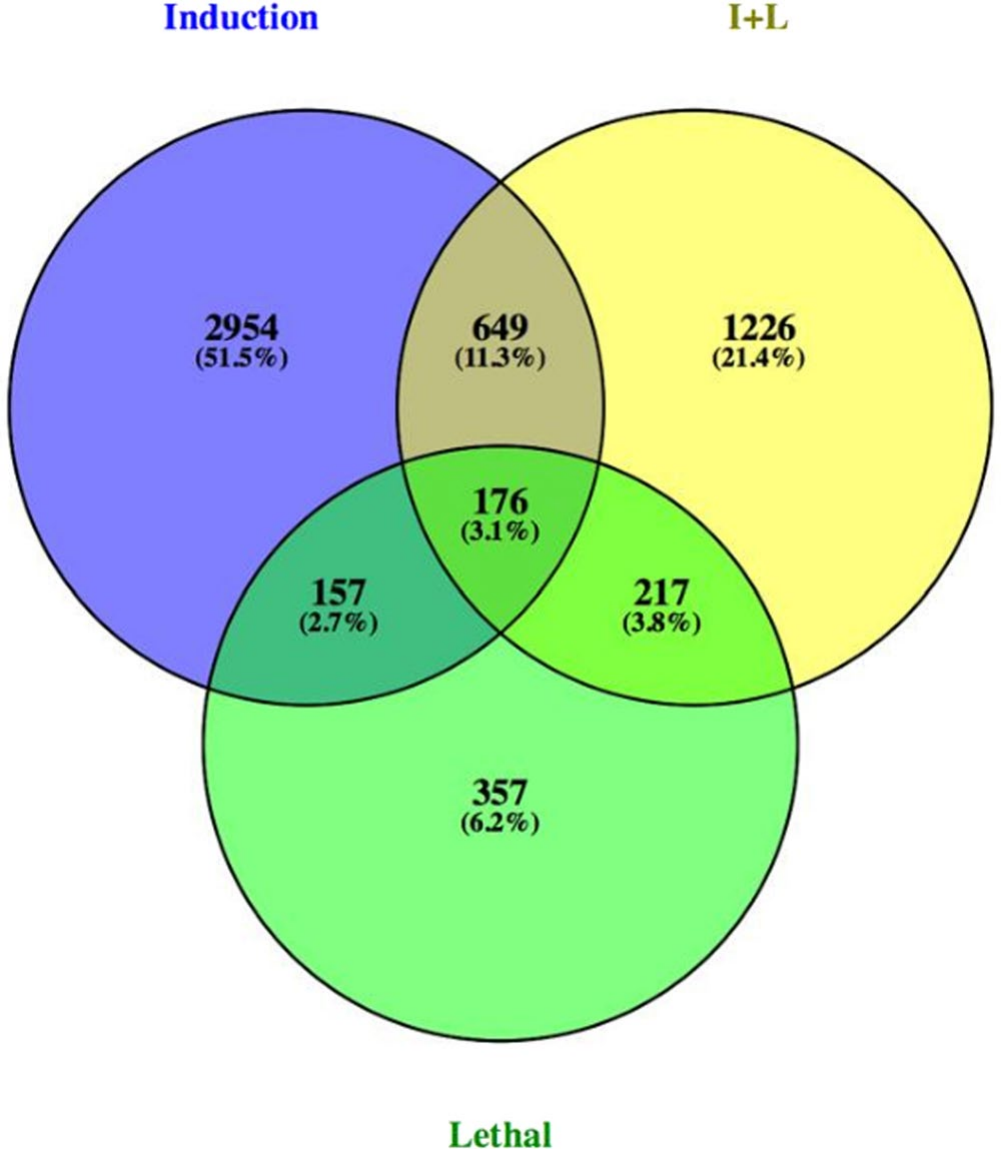
Venn diagram showing the number of overlapping *transcripts* that are differentially expressed under induction, I + L and lethal stresses.

The transcripts from different heat treatments were grouped based on their degree of expression (log2 fold change) values. Based on this, a heat map was developed, which showed transcript abundance levels in induction and I + L, and it was lower in lethal stress condition (Fig. 3). The transcripts which were highly expressed were also annotated using GFOLD software^19^ which is a reliable method to rank the differentially expressed genes (DGE). The GFOLD value for each gene can be considered as a robust fold change, which measures primarily the relative change of the expression level instead of the significance (i.e. P-value) of differential expression during Induction, I + L, Lethal stress (Supplementary File S2). The unigenes were sorted based on the fold change. The number of activated genes was more in induction stress (3936 transcripts) compared to I + L (2268 transcripts), and lethal stress (907 transcripts) (Fig. 4). The results show that during the induction stress, higher number of (3936) transcripts were significantly up-regulated which were early responsive genes for heat stress. There are many evidences to show that during different stress conditions, higher number and higher level of stress associated protein gene expression were observed in many crops in tolerant genotypes^2,18^ for abiotic stress.

### HSFs, HSPs and sHSP are altered during induction stress

It is very well known that HSP family plays an important role in heat stress^20^. We have examined the expression of different class of HSPs. Depending on the molecular weight they are classified into high molecular weight (HMW-HSP, 80–114 kDa), HSP70 (69–71 kDa), and low molecular weight (LMW-HSP, 15–30 kDa) HSPs. In the DGE, analysis thirty seven HSPs were activated during induction, twenty in I + L, and three under lethal stress conditions. These included both high and low molecular weight HSPs. The major HSPs activated were HSP90, HSP83, HSP70, low molecular HSPs like HSP17.1, HSP17.3, HSP17.4, HSP18.1, HSP18.6, HSP22, HSP26.5, HSP28. Majority of the HSPs were activated at early stages heat stress (during induction), and the number of transcripts decreased at higher level of stress (I + L). Further, only three HSPs were observed i.e the number of HSPs activated decreased in plants, which are directly exposed to lethal stress (Supplementary File S3). Hence, induction has enhanced expression of different HSPs. Therefore, the higher number and higher levels of expression during induction or acclimatization might be the key factor in acquired thermotolerance. It was observed in plant species and organism in tolerant genotypes that they have higher expression of HSFs and HSP in many abiotic stress^18,20^. These evidences and our results clearly show that the induction of HSFs and HSPs genes are the primary triggering factor in acquired thermotolerance.

Heat shock factors are involved in basal and acquired thermotolerance and play important roles in regulating the expression of heat shock proteins^20,21^. *HSF30* and *HSF1* were the two heat shock factors that were upregulated due to heat stress at induction and I + L (Supplementary File S3). It was reported that these HSFs were induced due to heat stress and were involved in the activation of HSPs expression in tomato^22,23^ and *Arabidopsis*^24^. In case of *Arabidopsis thaliana*, twenty-one HSFs were found and were classified into three major classes *viz* A, B, and C^25^. The class A and class B HSFs were found upregulated in our study (*HSFA2*, *HSFB*). Earlier studies on tomato, spinach and *Arabidopsis* indicates that HSFs are the key regulators under acquired thermotolerance^1^. In case of *Arabidopsis*, the overexpression of HSFs sustains longer expression of HSP^24^. This suggests that HSFs are crucial in regulating HSP expression thereby imparting thermotolerance.

### Protein modification under heat stress

Carbonylation is a commonly occurring protein oxidation phenomenon, which is activated by reactive oxygen species (ROS) production and lipid peroxidation^25^. A number of amino acids like proline, argenine, histidine, etc., gets oxidized to give out free carbonyl groups. The protein carbonyls act as an indication of ROS-mediated protein modification which is a useful tool in heat stress. Carbonylated proteins are repaired and removed from the system by many proteins like ATP synthase, 26 S proteasome subunit, glutathione synthetase, thioredoxin domain, E3 ubiquitin protein ligase, etc. Activation of these genes were observed during induction stress (Supplementary File S4) in banana. The upregulation of these genes during induction indicates the tissue, preparedness for minimizing protein modification during heat stress.

### Signal transduction and energy metabolism during high temperature stress

Hormone mediated signaling is also one of the major aspects in plants which regulates abiotic stress tolerance. Ethylene and ABA are known to be vital hormones in thermotolerance^26,27^. ABA signaling appears to be moe crucial in case of acquired thermotolerance, while ethylene signaling is more towards basal thermotolerance. At present, little is known about ABA involved in acquired thermotolerance. Although it is reasonable to hypothesize that similar genes might be induced by ABA during heat stress as during drought stress. Large numbers of stress- and ABA-regulated genes have been implicated in drought tolerance^27^, and specific transcripts have been shown to be induced by both heat and drought stress.

In confirmation with the above, in our study we have identified receptor-like kinase (RLK1) gene that are involved in ABA signal transduction^26^. RLK1 was found to be activated during induction (2.7 folds change) and I + L (1.7 folds change) (Supplementary File S5). In *Arabidopsis*, ABA mediated regulation was observed for stomata closure during abiotic stress response^27^, also in case of barley, where ABA was involved in the regulation of caryopsis under heat stress^14^. Here we have observed that ABA responsive transcription factors are involved in both induction and I + L thus, expanding our hypothesis that induction stress plays a major role acquired thermotolerance.

### Lipid metabolism during high temperature stress

Heat stress causes loss of membrane integrity and stability contributing to the decline in growth^28^. Thus stability and integrity are determined by the chemical composition of membrane (Phospholipids or fatty acids). Under optimal conditions the plant strikes a balance between producing and scavenging active oxygen species, which is observed to be disturbed in case of heat stress promoting the lipid peroxidation. Further, the lipid bilayer which is made up of fatty acids has both saturated and unsaturated forms. Thus maintenance of proper balance between these two forms is critical during heat stress condition^29^.

**Figure 5:**
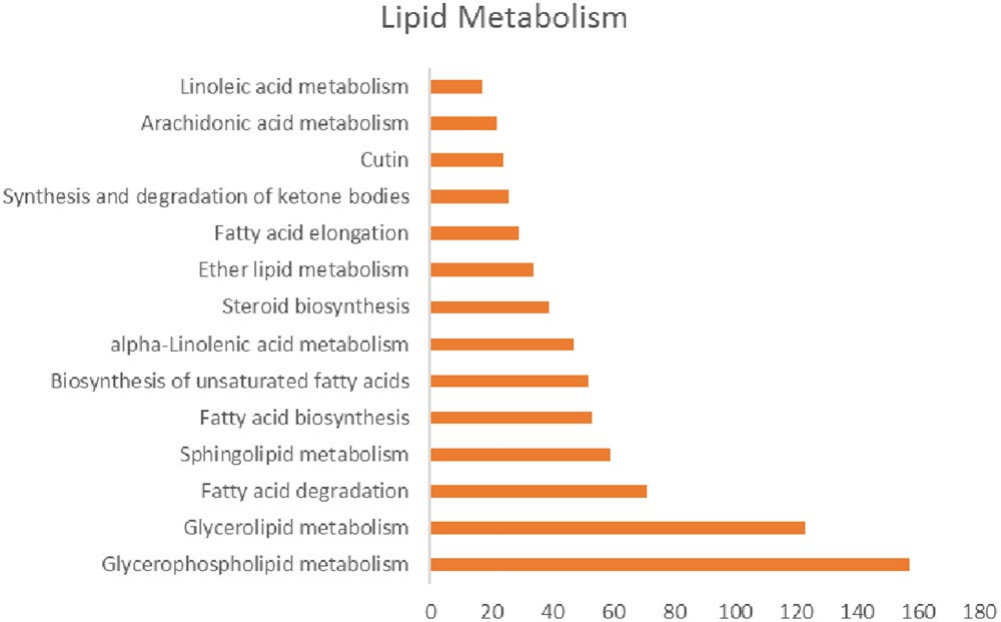
Lipid composition under heat stress.

In our study, the lipid metabolism was significantly affected during heat stress. We have observed the alteration of 753 unigenes belonging to 14 categories of lipid metabolism during heat stress (Fig. 5). They include glycerophospholipid metabolism, glycerolipid metabolism, fatty acid degradation, sphingolipid metabolism, fatty acid biosynthesis, Biosynthesis of unsaturated fatty acids, alpha-linolenic acid metabolism, steroid biosynthesis, ether lipid metabolism, fatty acid elongation, synthesis and degradation of ketone bodies, cutin, arachidonic acid and linoleic acid metabolism (Fig. 5). A total of 157 transcripts belongs to glycerophospholipid metabolism group were expressed during heat stress similar to results reported on maize leaves^30^. The transcripts TCONS_00021817 (glycerophospholipid biosynthetic process) and TCONS_00055753 (fatty acyl-CoA synthetase gene) were highly up-regulated during induction (I) and I + L stress (14 and 12 folds respectively) compared to lethal stress (L) (Supplementary File S6). The saturation of fatty acids helps in thermotolerance in plants^31^ and cell cultures^32^. The rise in temperature causes changes in lipid phase to form non-bilayer structures leading to disruption of membrane organization and photosystem II (PSII), under severe temperature conditions^33^. Thus the maintenance of membrane integrity is crucial to heat tolerance. This was achieved partly by activation of genes involved in the saturation of fatty acids during induction as observed in this study.

### Quantitative real time PCR validation from differentially expressed transcripts

**Figure 6:**
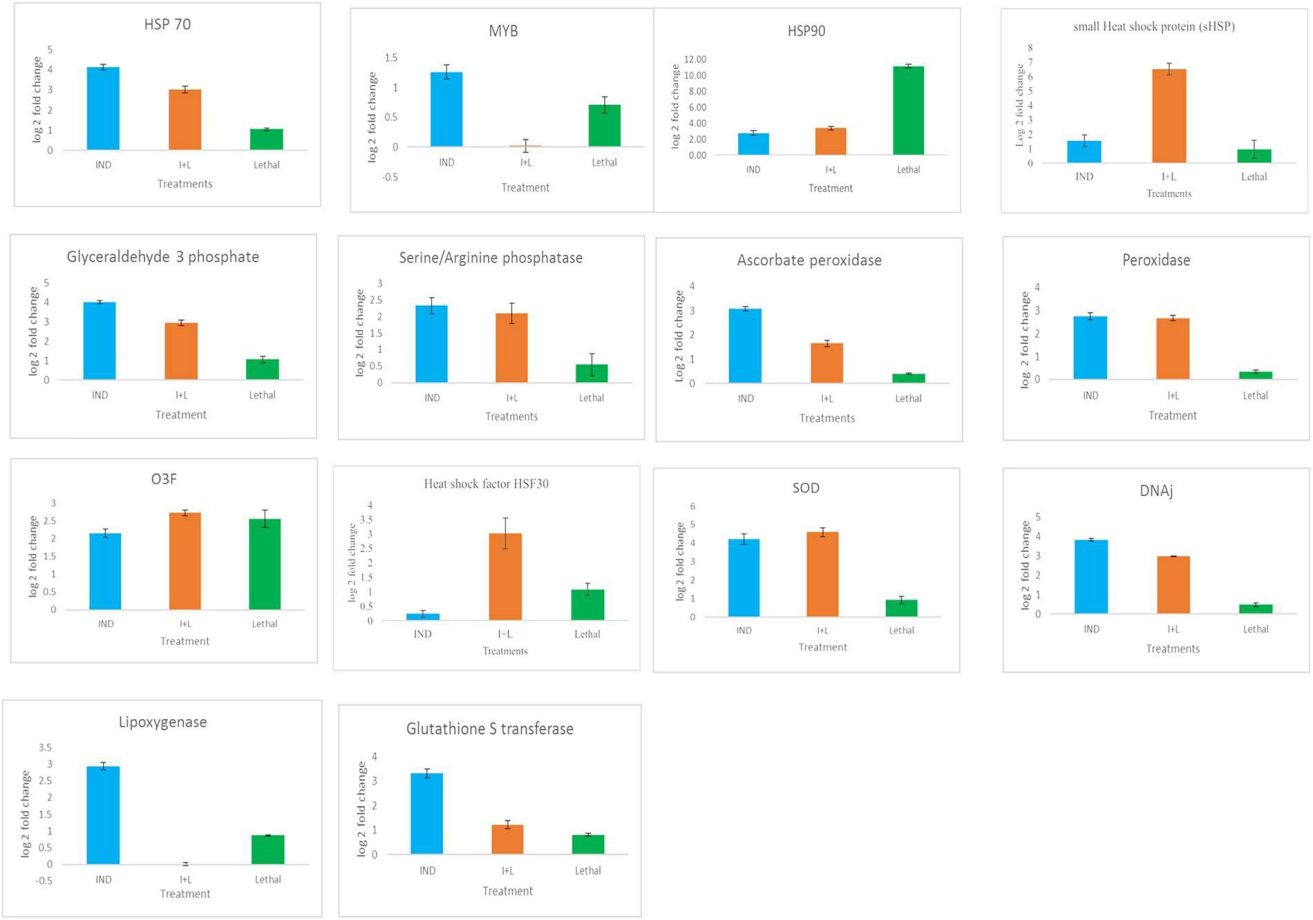
Expression levels of 12 genes after IND, I + L and Lethal stress were examined by qRT-PCR. The mRNA fold difference was relative to that of untreated control sample used as calibrator. Data are means ± SE of n = 3 independent experiments.

In order to validate differential gene expression results from transcriptome analysis, we have randomly selected fourteen genes for qPCR analysis. These fourteen genes were used and analyzed at four stages of stress treatment (Supplementary File S7). The genes used for qPCR analysis are: genes encoding heat shock proteins (HSPs): HSP90 and HSP70, small heat shock proteins (sHSP 17.1), heat shock factor (HSF30), genes involved in hormone regulation: serine/ threonine kinase involved in hormone metabolism, ROS enzymes like SOD, peroxidase, ascorbate peroxidase, other stress associated proteins: DNAj, omega-3 fatty acid desaturase which is involved in fatty acid biosynthesis, and transcription factor MYB. Here we observed twelve genes that were activated during induced condition, and I + L condition and two were down regulated compared to control (Fig. 6). HSP70, DNAj, serine/threonine kinase, glyceraldehyde-3-phosphate, glutathione S-transferase, peroxidase, omega-3- fatty acid desaturase, superoxide dismutase (SOD), HSP90, sHSP, HSF30 and ascorbate peroxidase, were upregulated while MYB and lipoxygenase were downregulated under heat stress (Fig. 6).

From qPCR study we observed 11 genes were activated during induction. Out of these, activity of ten genes were further enhanced during I + L. However, under lethal stress, the expression of nine genes were low when compared to I + L. Further, the correlation analysis between qPCR and RNA seq data was done using fold change expression levels. The higher correlation coefficient (r^2^ = 0.86, P < 0.001) indicates that the RNA-seq data generated is accurate, and can be used for expression profile analysis and for interpretation.

## Conclusion

In this study, high-throughput sequencing was used to characterize transcriptome of a non-model crop banana (cv. Grand Naine) to examine role of induction in acquired thermotolerance. The analysis of differentially expressed genes (DGE’s) under various stress conditions–induced (mild stress), induced followed by lethal stress, and lethal stress alone suggests that induction plays very important role in molecular and cellular preparedness for higher stress conditions. Induction also activates many genes, especially involved in the synthesis of heat shock proteins, antioxidants, hormone regulation, and other metabolic pathways which are known to impart thermotolerance. The mild or induction stress, followed by lethal stress (higher stress) is used to assess genetic potential and variability in various crops using temperature induction response (TIR) technique. Induction minimizes distortion in membrane integrity by activating genes in lipid metabolism. Recently, it has been proven that induction or priming of seedlings using mild stress will be of great adaptive advantage under natural growth conditions in many crops. Induction or acclimatization to stress is well evolved in plants. Due to vagaries of different stresses like drought, heat in nature, plants have evolved the acclimatization process which is initiated or triggered due to initial mild stress. Therefore, induction stress imparts acquired thermotolerance or heat stress memory in many crop species^34^. Thus, the induction stress technique can be widely adopted in crop species for imparting stress tolerance.

## Methods

### Plant materials and growth conditions

Tissue culture multiplied seedlings of banana (5–6 weeks old) cv. Grand Naine (*Musa* species AAA Group) with a uniform a growth stage were obtained from the University Of Agricultural Science (GKVK), Bengaluru, India. Young seedlings were kept under greenhouse conditions with relative humidity of 60% and temperature around 30 °C in polybags carrying red soil mixed with farm yard manure (FYM). Five leaf stage plants were allowed to acclimatize to normal conditions before subjecting them to heat stress. Plant growth was recorded and the effect of induction stress was measured (Z distribution analysis)^8^.

### Experimental design and heat stress treatment

The plants were subjected to various heat treatments *viz*, control, induction, induction followed by lethal, lethal^8^.

- Control: Seedlings maintained under normal temperature conditions at 30 °C (uninduced).
- Induction stress: Seedlings subjected to gradual increase in temperature from 30 °C–42 °C for 2 hours and 30 min.
- Induction + lethal: Seedlings after induction (from 30 °C–42 °C for 2 hours 30 min), were shifted to lethal stress–55 °C for 2 hours.
- Lethal stress: Un-induced (control plants) were directly shifted to 55 °C and kept for 2 hours.

The second leaf from the top was detached immediately after each stress/treatment (three biological replicates). Aliquots of the tissues were frozen immediately in liquid nitrogen and were stored at −80 °C until use.

### Total RNA isolation and Library Preparation

We have collected three replicates from different plant for each treatment. The RNA was extracted separately and later pooled. Total RNA was extracted from the leaf tissue samples using the RNeasy Plant extraction kit (Cat no: 74903, Qiagen, Germany). The concentration and quality of the samples were determined by a Nano Drop Spectrophotometer (Gene quant Pro, Amersham, Biosciences), and the integrity was determined using agarose gel electrophoresis (1.2% w.v). Following the manufacturer’s instructions of TruSeq RNA library protocol (Part # 15008136; Rev. A; Nov 2010) RNA libraries were prepared. The poly (A) mRNA was isolated from purified total RNA and fragmented for 2 minutes at elevated temperature (94 °C) in the presence of divalent cations and reverse hexamers. Second-strand cDNA was synthesized in the presence of DNA Polymerase I and RnaseH. The cDNA was cleaned up using Agencourt Ampure XP SPRI beads (Beckman Coulter). Illumina Adapters were ligated to the cDNA molecules after end repair and addition of ‘A’ base. SPRI cleanup was performed after ligation. The library was amplified using 8 cycles of PCR for enrichment of adapter ligated fragments. The prepared library was quantified using Nanodrop and validated for quality by running an aliquot on a High Sensitivity Bioanalyzer Chip (Agilent). The products were sequenced on NextSeq. 500 sequencer (Illumina) at the M/S Genotypic Technology, Bengaluru, facility.

### Processing and Mapping of Illumina reads

Reads were mapped to *Musa acuminata* (DH-Pahang) reference genome^5^ using softwares–TopHat^35^ (version 2.0), Cufflinks^36^ (version 2.0.1), and Bowtie2^37^ (version 2.0.5). TopHat is a fast splice junction mapper for RNA-Seq reads. It aligns RNA-Seq reads to genomes using the ultra-high-throughput short read aligner Bowtie, and then analyzes the mapping results to identify splice junctions between exons. Cufflinks assembles transcripts, estimates their abundances, and tests for differential expression and regulation in RNA-Seq samples. It accepts aligned RNA-Seq reads and assembles the alignments into a parsimonious set of transcripts. Cufflinks then estimates the relative abundances of these transcripts based on how many reads support each one, considering biases in the library preparation protocols. Cufflinks package was used to identify novel genes by comparing all the assembled transcripts to banana genome (http://banana-genome.cirad.fr/).

Those transcripts with a putative complete ORF were aligned to the NCBI nr (non-redundant) database and the Uni-Prot plant protein sequences (http://www.uniprot.org/uniprot/?query=taxonomy%3a33090&force=yes&format=fasta) by Blastx to find homologous proteins.

### Identification of differentially expressed genes

All unigenes were classified using Blastx software through homology search of rice (*Oryza sativa* japonica.), *Arabidopsis lyrata* (lyrate rockcress), *Cucumis sativus* (cucumber), *Fragaria vesca* (woodland strawberry), *Glycine max* (soybean), *Arabidopsis thaliana* (thale cress), *Solanum lycopersicum* (tomato), *Theobroma cacao* (cacao), *Vitis vinifera* (wine grape) genome in KEGG protein databases (E-value < 10^−5^). Later they were functionally annotated by Blast2GO Gene Ontology Functional Annotation Suit (E-value < 10^−5^) (http://www.blast2go.org/)^38^. Annocript software was also used to identify conserved domains (http://pfam.xfam.org/)^39^. The putative transcription factors were identified by searching Arabidopsis Gene Regulatory Information Server (AGRIS) Database (http://arabidopsis.med.ohio-state.edu/)^40^. GFOLD software was used to find the differentially expressed genes (http://www.tongji.edu.cn/~zhanglab/GFOLD/index.html)^19^. This software (generalized fold change) helps to produce biologically meaningful fold change values which were differentially expressed from the RNA-seq data. Based on the posterior distribution of log fold change GFOLD assigns a considerable statistical value for the expression change. GFOLD overcomes the shortcomings of P-value and fold change calculated by existing RNA-seq analysis methods and gives more stable and biological meaningful gene rankings when only a single biological replicate is available.Heat maps were designed and plotted using R package (https://bioconductor.org/packages/release/bioc/html/heatmaps.html).

### Validation of RNA-Seq by qRT-PCR

First-strand cDNA was generated from 1 μg of the total RNA used for RNA-Seq analysis (#K1622, Thermo Scientific, USA). The expression level of the target genes was determined by means of quantitative real-time PCR (qPCR) using ABI 7500 real-time PCR system (Applied Biosystems, Foster City, CA, USA) and DyNAmo Flash SYBR Green qPCR kit (#F-416L, Thermo Scientific). Primers for qPCR was designed using Integrated DNA Technologies (IDT)-oligo analysis tool online software (https://eu.idtdna.com/site) (Supplementary File S7). The reaction mixtures (20 μL) consisted of 2 μL of cDNA, 1 μL of each primer (5 pmole concentration), and 10 μL of SYBR mix. Thermal cycling conditions were 40 cycles of 95 °C for 15 sec, 60 °C for 30 sec (data collection point) and 72 °C for 15 sec followed by dissociation curve. Glyceraldehyde 3-phosphate dehydrogenase (GAPDH) was used as the reference gene^41^. The comparative CT method (2^−ΔΔct^) was used to quantify the relative expression of specific genes. The qPCR was carried out using three biological replicates and three technical replicates.

## Electronic supplementary material

Dataset 1

Dataset 4

Dataset 7

Dataset 2

Dataset 3

Dataset 5

Dataset 6
